# Secondary pulmonary alveolar proteinosis complicating myelodysplastic syndrome results in worsening of prognosis: a retrospective cohort study in Japan

**DOI:** 10.1186/1471-2466-14-37

**Published:** 2014-03-05

**Authors:** Haruyuki Ishii, John F Seymour, Ryushi Tazawa, Yoshikazu Inoue, Naoyuki Uchida, Aya Nishida, Yoshihito Kogure, Takeshi Saraya, Keisuke Tomii, Toshinori Takada, Yuko Itoh, Masayuki Hojo, Toshio Ichiwata, Hajime Goto, Koh Nakata

**Affiliations:** 1 Department of Respiratory Medicine, Kyorin University School of Medicine, 6-20-2 shinkawa, Mitaka-shi, Tokyo 1818611, Japan; 2 Department of Haematology, Peter MacCallum Cancer Centre, St Andrews Place, East Melbourne, Victoria 8006, Australia; 3 Bioscience Medical Research Center, Niigata University Medical & Dental Hospital, 1-754 Asahimachi-dori, Chuo-ku, Niigata 9518520, Japan; 4 Diffuse Lung Diseases and Respiratory Failure, Clinical Research Center, NHO Kinki-Chuo Chest Medical Center, 1180 Nagasone-cho, Kita-ku, Sakai, Osaka 5918555, Japan; 5 Department of Hematology, Toranomon Hospital, 2-2-2 Toranomon, Minato-ku, Tokyo 1058470, Japan; 6 Department of Respiratory Medicine, NHO Nagoya Medical Center, 4-1-1 Sannomal, Naka-ku, Nagoya, 4600001Japan; 7 Department of Pulmonary Medicine, Kobe City General Hospital, 4-6 Minatojimanakamachi, Chuo-ku, Kobe-city, Hyogo 6500046, Japan; 8 Division of Respiratory Medicine, Niigata University Graduate School of Medical and Dental Sciences, 1-754 Asahimachi-dori, Chuo-ku, Niigata 9518520, Japan; 9 Division of Respiratory Medicine, National Center for Global Health and Medicine, 1-21-1 Toyama, Shinjuku-ku, Tokyo 1628655, Japan; 10 Department of Respiratory Medicine, Tokyo Medical University Hachioji Medical Center, 1163 Tatemachi, Hachioji-shi, Tokyo 1930998, Japan

**Keywords:** Proteinosis, Myelodysplastic syndrome, GM-CSF, WPSS, Secondary pulmonary alveolar proteinosis, MDS, PAP, Refractory anemia

## Abstract

**Background:**

Secondary pulmonary alveolar proteinosis (sPAP) is a very rare lung disorder comprising approximately 10% of cases of acquired PAP. Hematological disorders are the most common underlying conditions of sPAP, of which 74% of cases demonstrate myelodysplastic syndrome (MDS). However, the impact of sPAP on the prognosis of underlying MDS remains unknown. The purpose of this study was to evaluate whether development of sPAP worsens the prognosis of MDS.

**Methods:**

Thirty-one cases of sPAP and underlying MDS were retrospectively classified into mild and severe cases consisting of very low-/low-risk groups and intermediate-/high-/very high-risk groups at the time of diagnosis of MDS, according to the prognostic scoring system based on the World Health Organization classification. Next, we compared the characteristics, disease duration, cumulative survival, and prognostic factors of the groups.

**Results:**

In contrast to previous reports on the prognosis of MDS, we found that the cumulative survival probability for mild MDS patients was similar to that in severe MDS patients. This is likely due to the poor prognosis of patients with mild MDS, whose 2-year survival rate was 46.2%. Notably, 75% and 62.5% of patients who died developed fatal infectious diseases and exacerbation of PAP, respectively, suggesting that the progression of PAP *per se* and/or PAP-associated infection contributed to poor prognosis. The use of corticosteroid therapy and a diffusing capacity of the lung for carbon monoxide of less than 44% were predictive of poor prognosis.

**Conclusion:**

Development of sPAP during the course of MDS may be an important adverse risk factor in prognosis of patients with mild MDS.

## Background

Pulmonary alveolar proteinosis (PAP), a rare disorder predominantly affecting the lungs, is characterized by accumulation of surfactant lipids and proteins in the alveoli and terminal bronchioles [1]. PAP is clinically classified into three distinct forms, namely, autoimmune, secondary, and congenital PAP [2]. Autoimmune PAP is associated with disruption of granulocyte/macrophage colony-stimulating factor (GM-CSF) signaling caused by high levels of GM-CSF autoantibody in the lungs [3]. Secondary PAP (sPAP) results from underlying diseases that presumably impair surfactant clearance because of abnormal numbers and functions of alveolar macrophages (AMs). Of the 40 cases of sPAP previously reported by our group [4], 88% (n = 35) involved hematological disorders as underlying diseases. The probability of survival at two years was 46% in cases with sPAP complicating hematological disorders. The median survival time for all cases including 17 patients who died within two years of the sPAP diagnosis was 16 months. Although myelodysplastic syndrome (MDS) is the most frequent underlying disease of sPAP (n = 26, 65%), little information is available on the prognostic impact of development of sPAP on patient outcome.

MDS, a group of clonal hematological stem-cell disorders with ineffective myeloid hematopoiesis and varying degrees of bone marrow failure, is associated with a significant risk of progression to acute myeloid leukemia (AML). Clinical manifestations are variable, from indolent conditions with near-normal life expectancy to forms that rapidly develop into AML [5]. Clarifying much of this heterogeneity, the World Health Organization (WHO) developed a classifications of MDS based on the presence of unilineage or multilineage dysplasia, bone marrow blast cell count, and cytogenetic features: refractory anemia (RA), RA with ringed sideroblasts (RARS), refractory cytopenia with multilineage dysplasia (RCMD), RCMD with ringed sideroblasts (RCMD-RS), RA with excess of blasts-1 (RAEB-1), and RA with excess of blasts-2 (RAEB-2) [6]. In 2007, Malcovati *et al.* developed a new prognostic scoring system (WHO classification-based prognostic scoring system (WPSS)) according to WHO subgroup, karyotype, and transfusion requirement. Through this system, cases with MDS are classified into very low-, low-, intermediate-, high-, or very high-risk groups [7]. WPSS is a dynamic system that accurately predicts the survival and risk of leukemic evolution in MDS patients at any time during the course of their disease. This time-dependent system seems particularly useful for lower-risk patients and for implementing risk-adapted treatment strategies.

Given the validation of WPSS criteria as a prognostic indicator for the course of MDS, it is applicable in prognosis evaluation of MDS complicated by PAP (MDS/sPAP). In the present study, we evaluated this issue for the first time and found that development of sPAP worsens the prognosis of patients with otherwise low-risk MDS.

## Methods

### Subjects

Thirty-one patients in Japan who were diagnosed with sPAP with underlying MDS from July 1999 to June 2013 were evaluated. We obtained agreement from all treating physicians for each identified case, according to the Guidelines for Epidemiological Studies by The Ministry of Health, Labour, and Welfare. This was a retrospective cohort study approved by the Ethical Board of Kyorin University (H23-085-01). Cases, part of which were reported previously, were identified retrospectively [8-18].

### Diagnosis

Diagnosis of MDS was made according to the 2002 WHO criteria [6]. One patient with unclassified MDS, two patients with myelofibrosis, and two patients with 20% marrow blasts who were considered as having AML were excluded from the study. MDS with isolated chromosome 5 deletion (del(5q)) and marrow blasts of <5% were included. Next, the patients were classified into RA, RARS, RCMD, RAEB-1, and RAEB-2 groups. Karyotypes were classified by using the International System for Cytogenetic Nomenclature Criteria [19]. Diagnosis of PAP was based on the following criteria: 1. histopathological findings from specimens obtained by surgical biopsy or transbronchial lung biopsy, or milk-like appearance with typical cytological findings from bronchoalveolar lavage fluid (BALF); 2. typical high-resolution computed tomography (HRCT) findings for PAP, such as ground glass opacity, consolidation, and interlobular septal thickening; and 3. negative result for serum GM-CSF autoantibody.

### Classification by WPSS

The WPSS score was calculated according to the method of Malcovati *et al.*[7] The score was calculated from the data of WHO subgroups (RA/RARS/5q–, RCMD/RCMD-RS, RAEB-1, and RAEB-2), karyotype abnormalities categorized according to the International Prognostic Scoring System [20], and transfusion requirement. The same weight (score of ≥1) was assigned to each variable for WHO subgroup, karyotype, and transfusion requirement. Based on the score, patients were classified according to the following five risk groups: very low (score **=** 0), low (score **=** 1), intermediate (score **=** 2), high (score **=** 3 to 4), and very high (score **=** 5 to 6). Transfusion dependency was defined as having at least one red blood cell (RBC) transfusion every eight weeks over a period of four months.

### Statistical analysis

The cumulative probability of survival and risk of progression to leukemia were estimated by using the Kaplan–Meier method. Patients undergoing transplantation treatment were censored at the time of the procedure in order to exclude any potential source of bias due to differential treatment. Variable data were analyzed through Kaplan–Meier methods to estimate the cumulative probabilities of overall survival. The difference in the cumulative probabilities within subcategories of patients was compared by using two-sided log rank test. Survival analyze was performed using Cox proportional model with time-dependent covariates to assess the effect of the variables of interest on overall survival.

Numeric data were evaluated for normal distribution and for equal variance by using the Kolmogorov–Smirnov test and Levene’s median test, respectively. Nonparametric data are presented as medians. Categorical data are presented as a percentage of the total or numerically, as appropriate. Statistical comparisons of nonparametric data were compared through the Wilcoxon test. Comparisons of categorical data were made with chi-square or Fischer’s exact tests. All tests were two-sided. Statistical significance was indicated by p values of <0.05. Data were analyzed by using SPSS 17.0 software for Windows.

## Results

**Figure 1:**
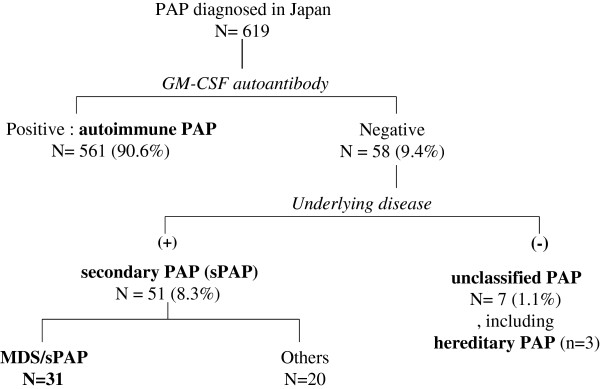
**Disposition of patients with pulmonary alveolar proteinosis (PAP) who were enrolled into this study.** Participants were stratified according to the presence or absence of granulocyte/macrophage-colony stimulating factor (GM-CSF) autoantibody, and then according to the presence or absence of an underlying disease known to cause PAP.

From September 1999 to May 2013, we centrally analyzed for GM-CSF autoantibody in the sera of 619 Japanese cases that had been diagnosed as PAP. Of those cases, 561 were positive for the antibody and 58 were negative. In the cases negative for GM-CSF antibody, 51 demonstrated obvious underlying diseases such as hematological disorders, autoimmune diseases, and infectious diseases. As shown in Figure 1, hematological disorders were the most common underlying disease, 31 cases of which involved MDS. These cases were investigated retrospectively in this study.

Demographic data are shown in Table 1. Diagnosis of MDS was performed in accordance with WHO criteria; 19, 1, 5, 3, and 3 cases were RA, RARS, RCMD, RAEB-1, and RAEB-2, respectively. Karyotyping revealed 2 cases of “high-riskes of g e, 24 cases of “intermediate-riskmediatef, and 5 cases of “low-risks disease. As previously reported [10], there was over-representation of trisomy 8, as it was present in 16 cases (51.6%). RBC transfusion dependency was observed in 11 cases.

**Table 1:** Demographic data at diagnosis of MDS in each group classified according to the WPSS

		**<WPSS risk groups>**		
		**<Very low + low >**	**<Inter - +high + very high>**	
**Median (min.–max.)**	**Total**	**Mild MDS**	**Severe MDS**	***p *** **value**
	**(n = 31)**	**(n = 13)**	**(n = 18)**	
Sex (M/F)	19/12	7/6	12/6	0.71
Age at Dx of MDS	50 (27–75)	45 (30–67)	50 (27–75)	0.54
HbG (g/dl)	9.4 (4.8–16.4)	11.4 (5.5–16.4)	9.0 (4.8–13.9)	0.06
ANC (× 10^9^/L)	1.84 (0.01–7.54)	1.46 (0.45–6.97)	2.74 (0.01–7.54)	0.68
PLT (× 10^9^/L)	65 (6–219)	45 (14–219)	69 (6–196)	0.31
WHO subgroup: n (%)				
RA/RARS	20 (65)	13 (100)	7 (39)	<0.001
RCMD	5 (16)	0 (0)	5 (28)	0.058
RAEB-1,2	6 (19)	0 (0)	6 (33)	0.02
Karyotype*: n (%)				
Good type	2 (7)	2 (15)	0	0.16
Intermediate type	24 (77)	11 (85)	13 (72)	0.66
Poor type	5 (16)	0 (0)	5 (28)	0.058
RBC transfusion dependency**: n (%)	11 (35)	0 (0)	11 (61)	<0.001

(*) Cytogenetics was as follows. Good type: normal, -Y, del(5q), del(20q); poor type: complex (≥ three abnormalities), chromosome 7 anomalies; and intermediate type: other abnormalities.

(**) RBC transfusion dependency was defined as having at least one RBC transfusion every eight weeks over a period of four months. ANC, absolute neutrophil count; Dx, diagnosis; HbG, hemoglobin; MDS, myelodysplastic syndrome; PLT, platelets; RA, refractory anemia; RAEB, refractory anemia with blasts; RARS, refractory anemia with ringed sideroblasts; RBC, red blood cell; RCMD, refractory anemia with multilineage dysplasia; WHO, World Health Organization; WPSS, WHO classification-based prognostic scoring system.

PAP was diagnosed on the basis of the BALF and HRCT results in 23 cases and by surgical biopsy and HRCT in eight cases. In 23 cases, diagnosis of MDS was done before diagnosis of PAP, whereas eight cases were diagnosed simultaneously.

According to the WPSS, the cases were classified into 2, 11, 7, 9, and 2 cases of very low-, low-, intermediate-, high-, and very high-risk groups, respectively. For statistical analysis, very low-/low-risk groups and intermediate-/high-/very high-risk groups were categorized as “mild MDS” and “severe MDS” DSere iz, respectively (Table 1). There was no difference in sex and age at the diagnosis of MDS and in hemoglobin concentration, absolute neutrophil count, and platelet count between mild and severe MDS cases. Then, the median age at diagnosis of MDS/sPAP was 51 years, and 84% of cases were symptomatic, with the most common symptoms being fever (45%), dyspnea on effort (42%), and cough (42%). The value of serum Krebs von den Lungen-6 (KL-6) and surfactant protein-D (SP-D) were elevated, and the diffusing capacities of the lung for carbon monoxide (%DLco) were very low in the absence of ventilation disorder in pulmonary function tests. There was no difference in the frequency of respiratory symptoms between patients with mild MDS and those with severe MDS. Serum biomarkers and pulmonary function tests showed no significant difference between the two groups.

**Figure 2:**
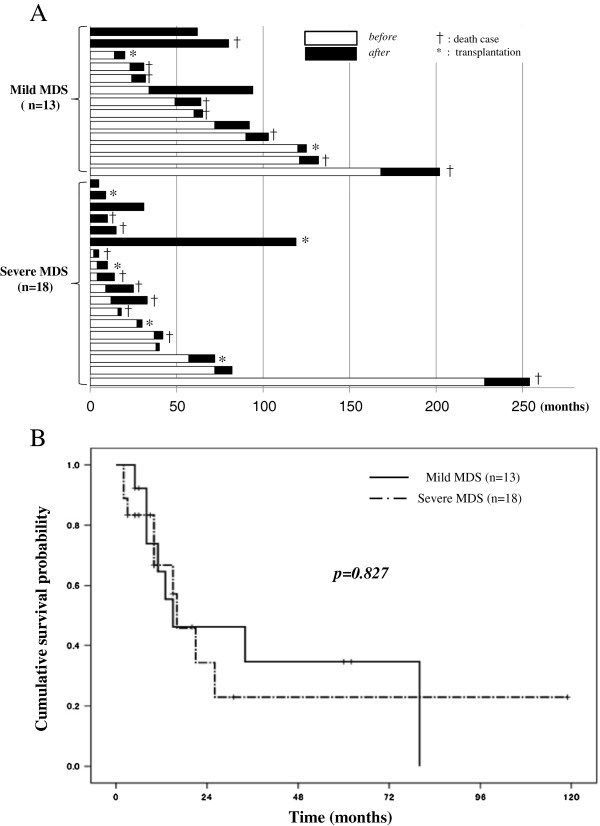
**Disease duration of secondary PAP in patients with MDS. A**: Duration of disease before or after the diagnosis of secondary pulmonary alveolar proteinosis (sPAP). The horizontal axis indicates the month after the diagnosis of myelodysplastic syndrome. The open column and closed column indicate the duration of disease respectively before and after the diagnosis of sPAP. The case that resulted in death is marked by † and the case that underwent transplantation therapy is marked by *. **B**: Cumulative survival probability after diagnosis of secondary PAP (sPAP) in patient groups with mild myelodysplastic syndrome (MDS) (n = 13) and severe MDS (n = 18). The horizontal axis indicates the month after the diagnosis of sPAP, and the vertical axis indicates the cumulative survival probability.

Follow-up periods after diagnosis of MDS ranged from five to 254 months (median, 40 months) in all patients (Additional file 1: Figure S1 and Additional file 2: Figure S2). During the follow-up period, 7 patients with mild MDS and 10 patients with severe MDS died, and 2 patients with mild MDS and 4 patients with severe MDS progressed to AML (Table 2). Two and five patients in the mild MDS and severe MDS groups, respectively, underwent transplantation therapies. They were censored at the time of the procedure. The duration from diagnosis of MDS to diagnosis of sPAP was variable, ranging from 0 to 168 months in the mild MDS group and from 0 to 228 months in the severe MDS group (Figure 2A). The median duration of MDS prior to diagnosis of sPAP in the mild MDS group was significantly longer than that in the severe MDS group (p = 0.034). Three patients in the mild MDS group and one in the severe MDS group survived for more than five years after diagnosis of sPAP. Of those, spontaneous remission of sPAP occurred in three cases.

**Table 2:** Clinical status at death (n = 17)

		**WPSS at diagnosis**	**WHO criteria at diagnosis of MDS**	**AML progression**	**Progression of PAP**	**Pneumonia**
	**No.**			**6 (35.3%)**	**11 (64.7%)**	**11 (64.7%)**
Mild MDS	1	Very low + low	RA			●
	2		RA		●	
	3		RA	●		●
	4		RA		●	●
	5		RA	●		
	6		RA		●	
	7		RA		●	
Severe MDS	8	Intermediate	RA			●
	9		RA		●	●
	10		RA		●	●
	11		RA	●	●	●
	12		RA	●		●
	13		RCMD		●	
	14	High + very high	RA		●	●
	15		RCMD	●	●	
	16		RAEB-1		●	●
	17		RAEB-2	●		●

AML, acute myeloid leukemia; MDS, myelodysplastic syndrome; PAP, pulmonary alveolar proteinosis; RA, refractory anemia; RAEB, refractory anemia with blasts; RCMD, refractory anemia with multilineage dysplasia; WHO, World Health Organization; WPSS, WHO classification-based prognostic scoring system.

A previous report [7] demonstrated that prediction of survival was dependent on the severity of MDS (as defined by WPSS) at any time of the disease. In contrast, patients with mild MDS in our study had cumulative survival probability similar to that of patients with severe MDS (Figure 2B, p = 0.827). This similarity may be due to poor prognosis of mild MDS after diagnosis of PAP. The cumulative survival probability curves of mild and severe MDS groups with median survivals of 13 and 15 months, respectively, are comparable. Concerning causes for the death of seven patients in the mild MDS group were progression to AML in two cases, PAP exacerbation in four cases, and fatal infectious disease in three cases (Table 2). Concerning causes for the death of 10 patients in the severe MDS group were progression to AML in 4 cases, PAP exacerbation in 7 cases, and fatal infectious disease in 8 cases. Fatal infectious diseases consistently arose from severe pneumonia with (n = 4) or without systemic sepsis, suggesting that progression of PAP was the major cause of death in both mild and severe MDS patients. These results suggest that occurrence of sPAP principally reduced the survival of patients with mild MDS. Pathogens isolated in the fatal cases were identified as *Aspergillus* species (four cases), *Pseudomonas aeruginosa* (four cases), and non-tuberculosis *Mycobacterium* species (four cases).

**Figure 3:**
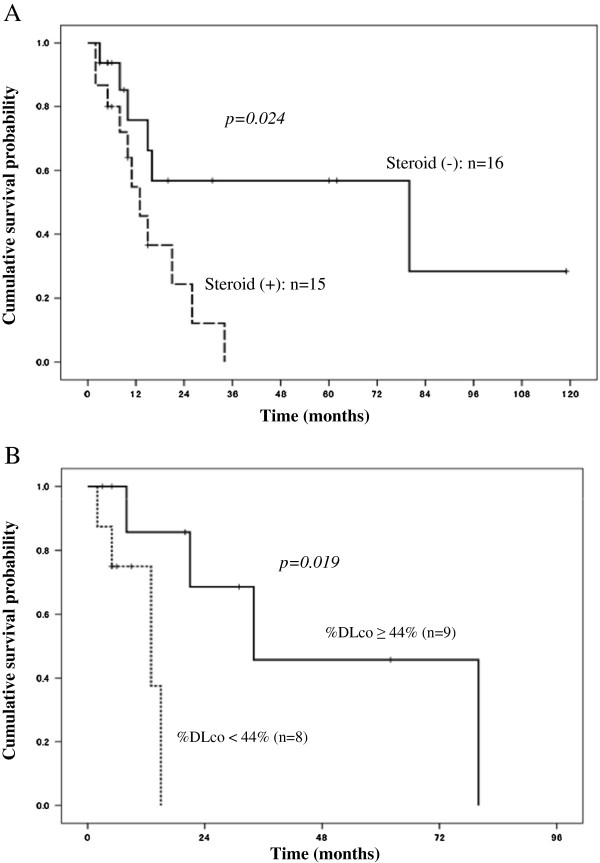
**Risk factors for the prognosis of secondary PAP in patients with MDS. A**: Cumulative survival probability after diagnosis of secondary PAP (sPAP) in myelodysplastic syndrome (MDS) cases with steroid therapy (n = 15) and in MDS cases without steroid therapy (n = 16). The horizontal axis indicates the month after the diagnosis of sPAP, and the vertical axis indicates the cumulative survival probability. The number of cases that received steroid therapy in the mild and severe MDS groups were six (46%) and nine (59%), respectively. **B**: Cumulative survival probability after diagnosis of secondary PAP (sPAP) in myelodysplastic syndrome (MDS) cases with diffusing capacity of the lung for carbon monoxide (%DLco) of <44% (n = 8) and in MDS cases with%DLco of >44% (n = 9). The horizontal axis indicates month after the diagnosis of sPAP, and the vertical axis indicates the cumulative survival probability.

By performing univariate analysis using Cox proportional model we then searched for potential prognostic factors. Age, sex, respiratory symptoms, history of respiratory failure, diagnostic procedure for sPAP, and MDS group (mild or severe), were not associated with survival at the time of diagnosis of sPAP (Table 3). Treatment with corticosteroids was associated with poor survival (p = 0.024) (Figure 3A). However, the number of patients treated with steroid therapy did not differ between mild and severe MDS groups. A %DLco of <44% (Figure 3B) predicted poor prognosis (p = 0.019), whereas %vital capacity (%VC), forced expiratory volume (FEV) 1.0%, serum KL-6, SP-D, and surfactant protein-A (SP-A) did not.

**Table 3:** Univariate analysis of overall survival after diagnosis of sPAP in MDS

**Variable at diagnosis of sPAP**	**(n)**	**75% of OS (months)**	**50% of OS (months)**	**HR (95% CI)**	***P *** **value**
Age: 51 yrs or younger	16	8	16		
Older than 52 yrs	15	10	15	1.29 (0.48-3.41)	0.607
Gender: Male	19	10	16		
Female	12	11	21	1.12 (0.43-2.94)	0.804
MDS group: mild	13	10	15		
severe	18	11	16	1.11 (0.42-2.95)	0.830
Symptoms: (-)	5	26	26		
(+)	26	6	15	1.50 (0.33-6.65)	0.592
Dx procedure: Bronchoscopy	23	11	13		
Surgical biopsy	8	15	26	0.69 (0.23-2.04)	0.507
Respiratory failure: (-)	21	11	26		
(+)	10	5	10	2.18 (0.77-6.22)	0.142
Use of corticosteroid: (-)	16	16	80		
(+)	15	10	13	3.20 (1.09-9.38)	0.034
Serum KL-6 (U/ml): < 1960	16	13	26		
1960 ≦	15	5	15	1.52 (0.58-4.00)	0.389
Serum SP-D (ng/ml): < 147	15	10	26		
147 ≦	15	8	15	1.80 (0.62-5.22)	0.278
Serum SP-A (ng/ml): < 79	16	11	26		
79 ≦	14	5	15	2.79 (0.965-8.06)	0.058
%VC: 87 ≦	11	15	34		
< 87	11	8	15	3.27 (0.79-13.52)	0.101
FEV1%: 86 ≦	12	8	16		
< 86	10	13	34	0.52 (0.14-1.87)	0.322
%DLco: 44 ≦	9	21	34		
< 44	8	5	13	9.98 (1.03-96.11)	0.046

CI indicates confidence interval; OS, overall survival; DLco, diffusing capacity of the lung for carbon monoxide; Dx, diagnosis; FEV, forced expiratory volume; HR, hazard ratio; KL-6, krebs von den lungen-6; MDS, myelodysplastic syndrome; SP-A, surfactant protein -A; sPAP, secondary pulmonary alveolar proteinosis; SP-D, surfactant protein -D; VC, vital capacity.

## Discussion

For the first time, we evaluated the prognosis of MDS/sPAP in a substantial cohort of patients, comparing mild and severe MDS as classified according to WPSS criteria. Our data demonstrate that the duration from diagnosis of MDS to diagnosis of sPAP was longer in mild MDS than that in severe MDS, but the survival probability was similar after the diagnosis of sPAP regardless of MDS severity. As a whole, occurrence of PAP appeared to worsen the prognosis of patients with mild MDS. This result is supported by the fact that the major cause of death was not MDS-associated but rather sPAP-associated respiratory failure or infections. Prior to our report, 21 cases with MDS/sPAP have been reported [8-18]. Most reports [9-18,21-24] describe a single case, whereas only two publications report multiple cases [8,25]. The clinical course described in these reports suggest a poor prognosis for MDS/sPAP patients, but no prior report clearly quantifies the outlook for these patients and compares this to a predicted outcome by using validated prognostic scores such as WPSS. MDS/sPAP is so rare a disease that neither pulmonologists nor hematologists encounter such patients very often. In fact, in more than 10 years, 2 to 5 cases were diagnosed as MDS/sPAP annually in our analyses for serum GM-CSF autoantibody in over 600 diagnosed PAP cases from all over Japan; thus, we have finally reached cumulative 31 cases with MDS/sPAP.

According to the WHO criteria, 20 of the 31 cases were RA/RARS, 5 cases were RCMD, and 6 cases were RAEB1-2. The proportion of the number for each subtype in the total number of cases was comparable to literature data in terms of frequency and subtype distribution, suggesting that the risk of PAP complicating MDS is similar regardless of the subtype of MDS. It is speculated that AMs in patients with MDS derive from abnormal bone marrow precursor cells and are defective in both surfactant homeostasis and host defense, hence the progression of PAP and PAP-associated infections even in cases with mild MDS. Previous studies reported that in the absence of complicating sPAP, the five-year survival probability for patients with RA and RARS was 74% [26], whereas our cases with RA plus RARS had substantially inferior prognosis (0.69) (Additional file 2: Figure S2).

It is noteworthy that treatment with corticosteroids was associated with a markedly inferior prognosis. In Japan, steroid therapy often has been used for PAP but no evidence of its efficacy has been found. To our surprise, 15 of 31 cases had undergone steroid therapy during the course of sPAP. Our data reveal that steroid-treated patients had worse prognosis than did patients without steroid therapy. Given that the predominant cause of death was infective complications potentially exacerbated by steroid-related immunosuppression, these data clearly caution against the use of steroid therapy in such patients.

Treatment of MDS/sPAP should be directed toward the underlying malignancy, i.e., MDS. It should also aim at restoring hematopoietic function, either through allogeneic bone marrow transplant, which has curative potential for both MDS and sPAP, or through hypomethylating agent therapy for MDS, which can restore numerical hematologic parameters. However, functional cellular defects will likely remain, as such therapy does not necessarily eradicate the underlying clone, but rather enhances cellular differentiation [27,28]. Nevertheless, seven cases with MDS/sPAP to date in our cohort had undergone transplantation therapy. Of those, three patients died of pneumonia within three months of the transplantation therapy. Therefore, we do not have any convincing evidence to recommend transplantation therapy in the early stages of the disease. Although whole-lung lavage and segmental bronchial lavage were performed in 10 patients, only 3 cases showed the efficacy of lung-lavage therapy.

Infection often coexists with MDS/sPAP, although the causal relationship between PAP and infection is not clear. Superimposed infection accounts for a significant degree of morbidity and mortality in patients with sPAP. In the present study, 11 among 17 cases with fatal outcomes developed fatal infectious diseases. Considering that this complication was observed in the mild MDS and severe MDS groups, pneumonia accompanied with sPAP might be the trigger of fatal infection. Nevertheless, the present number of cases (31) is too small for accurate prognosis evaluation of MDS/sPAP; future international collaboration may be necessary to overcome this difficulty.

## Conclusions

Complication of sPAP is an important risk factor in the prognosis of MDS. We believe that the present data will contribute to the management and treatment of the disease.

## Abbreviations

AM: Alveolar macrophage; AML: Acute myeloid leukemia; ANC: Absolute neutrophil count; BALF: Bronchoalveolar lavage fluid; CEA: Carcinoembryonic antigen; CT: Computed tomography; DLco: Diffusing capacity of the lung for carbon monoxide; Dx: Diagnosis; FEV: Forced expiratory volume; GM-CSF: Granulocyte macrophage colony-stimulating factor; HbG: Hemoglobin; HRCT: High-resolution computed tomography; KL-6: Krebs von den Lungen-6; MDS: Myelodysplastic syndrome; PAP: Pulmonary alveolar proteinosis; PLT: Platelets; RA: Refractory anemia; RARS: Refractory anemia with ringed sideroblasts; RAEB: Refractory anemia with blasts; RBC: Red blood cell; RCMD: Refractory anemia with multilineage dysplasia; SD: Standard deviation; SP-A: Surfactant protein-A; sPAP: Secondary pulmonary alveolar proteinosis; SP-D: Surfactant protein-D; VC: Vital capacity; WHO: World Health Organization; WPSS: WHO classification-based prognostic scoring system.

## Competing interests

Financial/non-financial competing interests

The authors report no potential conflicts of interest with any companies or organizations whose products or services are mentioned in this article.

## Authors’ contributions

The authors take responsibility and vouch for the completeness and accuracy of the data and analyses. HI and KN are the guarantors of the entire manuscript. HI and JS contributed to the study concept and design, coordination of data acquisition, and writing of the article. RT, YI, NU, AN, YK, and TS contributed to the data analysis. KT, TT, YI, MH, TI, and HG contributed to the interpretation of the data and writing of the article. KN participated in writing and critically revising the manuscript. All authors read and approved the final manuscript.

## Pre-publication history

The pre-publication history for this paper can be accessed here:

http://www.biomedcentral.com/1471-2466/14/37/prepub

## Supplementary Material

Additional file 1: Figure S1 Survival curves after diagnosis of MDS in each mild and severe MDS. Click here for file

Additional file 2: Figure S2 Survival curves in each MDS groups classified by WHO-criteria. Click here for file
