# Supplementary figures and images for: Secondary pulmonary alveolar proteinosis complicating myelodysplastic syndrome results in worsening of prognosis: a retrospective cohort study in Japan
Source: BMC Pulm Med. 2014 Mar 5;14:37. doi: 10.1186/1471-2466-14-37 (PMC3946190; doi:10.1186/1471-2466-14-37)

**< Additional Figure-1 >**

*Survival curves after diagnosis of MDS in each mild and severe MDS*

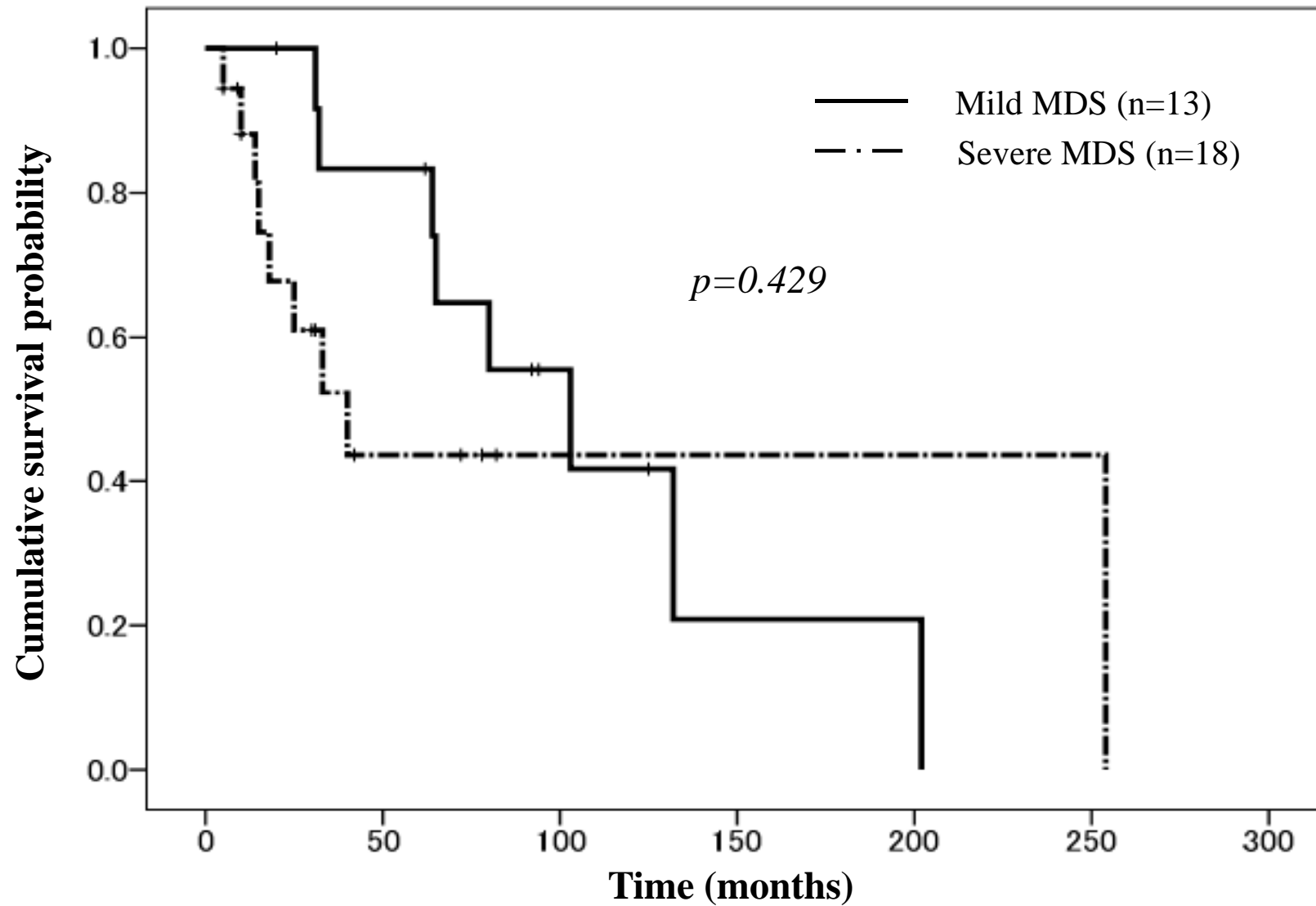

Supplement: Additional file 1: Figure S1 — Survival curves after diagnosis of MDS in each mild and severe MDS. [file 1471-2466-14-37-S1.pdf]

**< Additional Figure-2 >**

*Survival curves in each MDS groups classified by WHO-criteria*

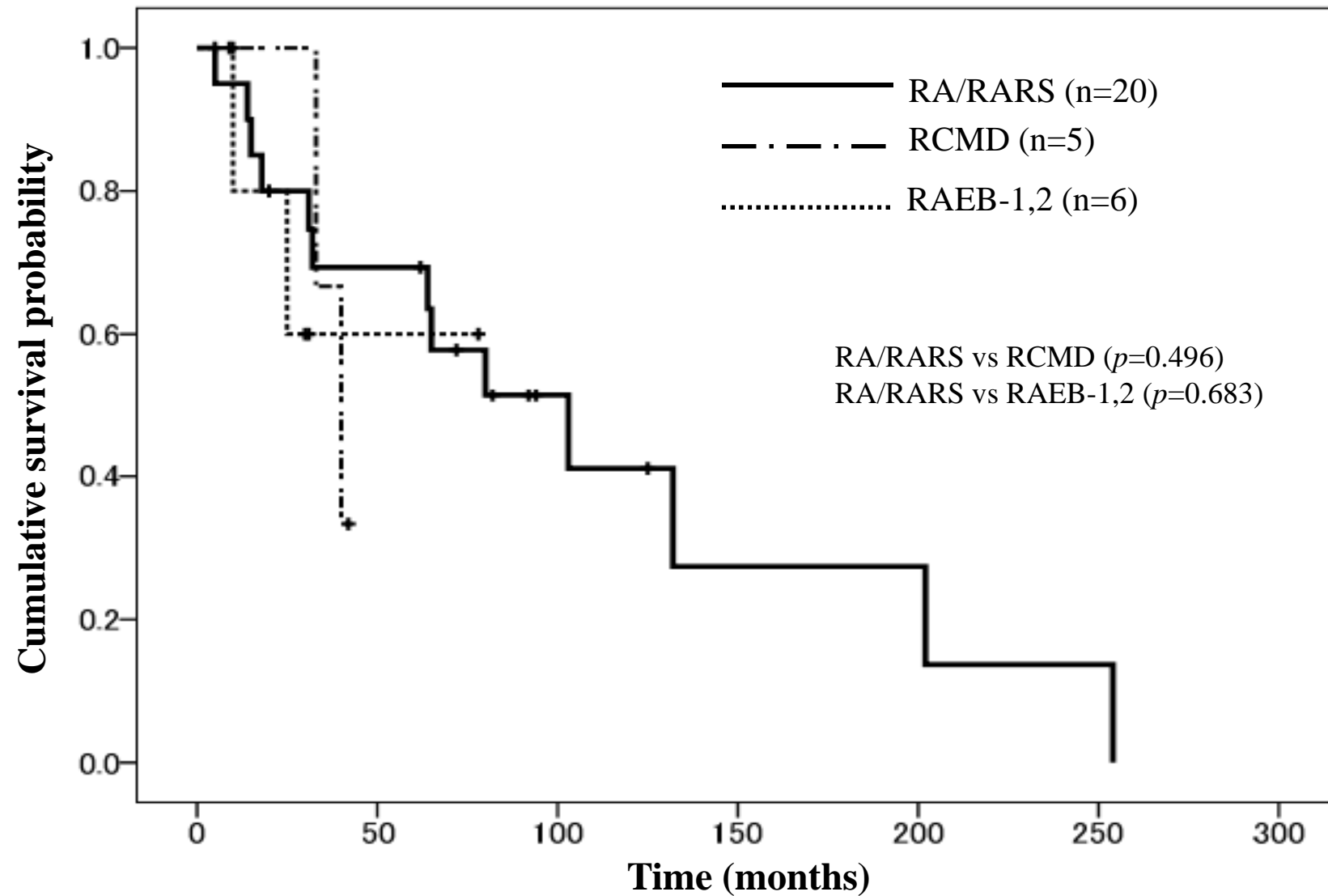

Supplement: Additional file 2: Figure S2 — Survival curves in each MDS groups classified by WHO-criteria. [file 1471-2466-14-37-S2.pdf]
